# OntoSpreadEd: Developing Ontologies with a Dedicated Online Template Spreadsheet Editor

**DOI:** 10.12688/wellcomeopenres.24408.1

**Published:** 2025-07-25

**Authors:** Björn Gehrke, Maya Braun, Paulina Schenk, Robert West, Susan Michie, Janna Hastings

**Affiliations:** 1 Institute for Implementation Science in Health Care, University of Zurich Faculty of Medicine, Zürich, Zurich, Switzerland; 2 Institute of Computer Science, Osnabrück University, Osnabrück, Lower Saxony, Germany; 3 Department of Experimental Clinical and Health Psychology, Ghent University, Ghent, Flanders, Belgium; 4 University College London Centre for Behaviour Change, London, England, UK; 5 University of St Gallen School of Medicine, St. Gallen, St. Gallen, Switzerland

**Keywords:** ontology, ontology editor, knowledge representation, knowledge formalisation, ontology development

## Abstract

**Background:**

Ontologies are machine-readable representations of knowledge that can accelerate research across various disciplines. To build ontologies, domain scientists must formally capture their knowledge of their domains in structured forms. However, existing ontology editing tools are often built for ontologists or other technical experts who are familiar with the underlying Web Ontology Language (OWL) and are comfortable working with technically dense interfaces, or even without a visual interface. The resulting technically oriented tools create barriers to adoption and use by nontechnical domain experts.

**Methods:**

To address this challenge, we present the Ontology Spreadsheet Editor (OntoSpreadEd), a web-based spreadsheet-like editor for ontologies that aims to make ontology development more efficient and accessible to a wide range of users. OntoSpreadEd is a web application that utilizes GitHub as a central version control platform, allowing users to create, edit and review ontologies using a familiar spreadsheet interface. The editor features a range of useful features, including autocomplete, validation and searching, to facilitate the development process.

**Conclusion:**

OntoSpreadEd offers an accessible approach to ontology development that can help bridge the gap between researchers and ontology editors, accelerating the adoption of ontologies across diverse research fields.

## 1 Introduction

An ontology is a machine-readable way of representing knowledge about things in the universe that we want to represent (e.g. objects, processes or qualities). This usually includes unique identifiers, labels and definitions as well as well-defined relationships between them ( Arp *et al.*, 2015). It is a powerful tool with the potential to accelerate research by (1) providing a shared vocabulary, (2) structuring existing knowledge and (3) facilitating computer-driven methods for a variety of applications, including evidence synthesis, data analysis and decision support ( National Academies of Sciences and Medicine *et al.*, 2022). Ontologies have been successfully used to improve and accelerate research, with perhaps the most prominent example being the Gene Ontology (GO) ( The Gene Ontology Consortium, 2019), an ontology that maps the function of genes and gene products. It provides an open searchable database that is updated regularly, has been used to annotate more than 100,000 peer-reviewed publications and has facilitated insights that would not be possible without the ontology. The Gene Ontology has been cited more than 40,000 times, demonstrating its immense influence in the field of biology.

This example demonstrates the potential impact of an ontology on a research field. Within the biological and biomedical sciences, many well-curated high-quality ontologies can be found in the repository of the Open Biological and Biomedical Ontology Foundry ( Smith *et al.*, 2007), a community platform for the broader domain.

In recent years, ontologies have started to be adopted within the behavioural and social sciences ( Hastings *et al.*, 2024). Most prominently, the Behaviour Change Intervention Ontology (BCIO) has pioneered the use of ontologies in this domain ( Michie *et al.*, 2021). The BCIO aims to represent the different aspects of behavioural interventions, such as techniques used in the intervention, their delivery, target behaviour and target mechanisms. They also represent their context, primarily the intervention setting and the target population. Such an ontology can be used to integrate evidence across and beyond the behavioural and social sciences.

### 1.1 Web Ontology Language

Many ontologies are written in the Web Ontology Language (OWL), which allows the representation of knowledge about things that exist within a particular domain and their interrelations. It provides a formal framework that supports automated reasoning and facilitates data linkage and alignment across various domains, academic disciplines, applications and systems.

OWL refers to anything it defines as an *entity*. There are different types of entities: representations of things that exist are termed *classes* and relations between classes are called *object properties*, while *data properties* link a class to basic data types, such as text or numbers. For example, the BCIO defines the *class* “behaviour change intervention”, which is connected via the *object property* “has process part” to the *class* “behaviour change intervention content”. In the Contextualised and Personalised Physical activity and Exercise Recommendations (COPPER) ontology ( Braun *et al.*, 2025), the *data property* “hasMinimumDurationInMinutes” is, among others, used to state that “climbing” is a “physical performance behaviour” which lasts for at least five minutes. In addition, OWL offers *annotation properties* to provide additional information about an entity, such as its label, description or author, without affecting the logical structure of the ontology. For example, the BCIO *class* “behaviour change intervention” has a textual definition via the *annotation property* “definition”.

Typically, OWL ontologies form a hierarchy where, in the simplest case, each class has a parent class, which is formalised in OWL as each class being a *sub-class* of another class. However, classes can also be expressed in terms of other classes using complex expressions, such as the previously mentioned expression associated with the class “climbing”. Automatic reasoning tools can then analyse and reorganise the consequent complex hierarchy and check for inconsistencies or logical errors within the ontology.

Each entity (e.g. class, object property and data property) in OWL is uniquely identified by an *IRI* (Internationalized Resource Identifier), a browsable address on the Internet with support for a wide range of characters, including those from different languages. Because an IRI can be long, it is often shortened to a short identifier known as *CURIE*. 

For example, the IRI http://purl.obolibrary.org/obo/BFO_0000001 can be shrunk to the CURIE BFO:0000001 (assuming a prefix BFO is defined as http://purl.obolibrary.org/obo/BFO_). Furthermore, it is recommended to always annotate OWL entities with a *label* for human readability.

### 1.2 Tools to develop and evaluate ontologies

Ontologies are not yet widely used in the behavioural and social sciences due in part to a lack of tools to support their development and maintenance.

Ontologies are typically developed in OWL and made available as a single file online for download. As this is usually a shared effort by multiple people, ontology development is often performed using *version control systems*. These systems can keep track of individual edits that might be made at the same time while enabling them to be merged if needed, thereby preventing version conflicts and ensuring that all developers have access to the same version. Platforms such as GitHub ^1^ and GitLab ^2^ add additional functionalities for reviewing changes, publishing releases (i.e. new versions) and systems for raising and discussing issues. Tools for ontology development may directly or indirectly integrate version control systems to ease the development process of ontologies.

Previous qualitative research with ontology developers from different domains and using a variety of editors has identified a number of common barriers related to ontology development and evaluation. These include a lack of support for searching across multiple ontologies, visualisation, reasoning, debugging and evaluation ( Vigo *et al.*, 2014). Tools for ontology development and editing can be challenging to use for those that are unfamiliar with them and have interfaces that tend to be unintuitive ( Mendonça *et al.*, 2021). We argue that this is especially the case for users in the behavioural and social sciences, where researchers do not frequently need to use these kinds of tools and therefore do not have the experience to help them learn to use the tools.

One of the most prominent ontology-editing tools is Protégé. Protégé is a free, open-source ontology editor created by the Stanford Center for Biomedical Informatics Research (BMIR) ( Musen & Protégé Team, 2015), which started in the 1980s and is frequently updated. It has a desktop system (currently Protégé 5) and a web-based system (WebProtégé), both being graphical editors for OWL files, though it exports to various other formats. Although similar tools to Protégé exist, such as the web-based onto4all editor ( Mendonça *et al.*, 2021) ^3^, Protégé is the most widely used ( Warren, 2013) and popular within research, as well as in companies and government institutions. Despite its long existence and comparatively high usability evaluations in multiple studies ( Malik, 2017; Musen & Protégé Team, 2015; Siricharoen, 2018; Warren, 2013), it remains difficult to use for many. Using Protégé requires expertise with OWL terminology and structure, making it more difficult for researchers with domain knowledge but less technical expertise. This also makes the review and curation of ontologies more challenging.

Protégé does not have built-in integration of a version control system, creating additional barriers when multiple users wish to maintain an ontology jointly. To overcome some of these pitfalls, *ROBOT* (self-referencing acronym for ***R**OBOT is an **OBO** **T**ool*) was developed. *ROBOT* is an open-source library and scripting tool that can automate many ontology development, merging and editing tasks that would otherwise be carried out in an ontology editor, such as Protégé. This tool was created to automate ontology workflows ( Jackson *et al.*, 2019), making ontology development more efficient. It allows a majority of the work in ontology development to be done in spreadsheets, which are more accessible to researchers across disciplines compared to the graphic interface of Protégé and similar editors. However, ROBOT still requires the use of the command-line and additional scripts to integrate with other OWL tools and version control systems. As such, it is still not sufficiently accessible to researchers within the behavioural and social sciences.

To the best of our knowledge, there are no ontology editors available that are user-friendly for researchers who do not have advanced technical expertise while simultaneously allowing for version control integration.

### 1.3 Aims of this paper

This study aims to address the lack of accessible tools for ontology development by introducing OntoSpreadEd, a spreadsheet-based ontology editor that integrates a version control system. This tool is designed to bridge the usability gap between domain experts, especially in the behavioural and social sciences, and traditional ontology development environments that require advanced technical skills.

## 2 Requirements

OntoSpreadEd was developed for and has successfully been used in several ontology development projects within the behavioural and social sciences, including the Human Behaviour-Change Project ( Michie *et al.*, 2021), the Addiction Ontology ( Hastings *et al.*, 2020) and the GALENOS project ( Schenk *et al.*, 2024). Throughout these projects, it has more than 20 users with a background in behavioural or social sciences, typically psychology, with varying amounts of technical expertise. OntoSpreadEd was developed iteratively throughout these projects to improve its usability. OntoSpreadEd currently offers a range of functionalities to users.

Throughout its use, key requirements of the tool have become apparent. Most importantly, the tool must provide an **accessible spreadsheet-based collaborative working environment** with an intuitive user interface for non-technical users. This means that users must be able to **open and edit spreadsheets** and save them to a repository. Here, users should be able to add or edit classes, their definitions and, if applicable, other relationships. They should also be able to leave **additional notes and annotations** in specified columns. To make this as usable as possible, the tool should support familiar spreadsheet formatting (e.g., conditional highlighting) and filtering to streamline data entry. The tool then needs to be able to **automatically convert spreadsheets to OWL**. As reuse of entities is the gold standard in ontology development ( Smith *et al.*, 2007), the tool also needs to be able to specify ontologies to reuse and how to reuse them.

Throughout its usage, further requirements have emerged. First, **human error** must be avoided or detected and corrected as much as possible throughout the development of the ontology. To achieve this, the tool should identify and flag sources of potential errors in converting spreadsheets to OWL as early as possible. For this, autofill should be used to find previously defined entities more easily, and validation tests should be carried out whenever a spreadsheet is saved and whenever conversion to OWL is initiated. Second, complex ontologies often consist of **more than one spreadsheet**. In the tool, we needed to be able to add multiple spreadsheets, define their relationships and automatically merge the resulting OWL files into one ontology. Third, **additional ontology exploration** opportunities would allow users to navigate more easily through more complex ontologies that include multiple spreadsheets. To allow for this, both a search tool and a visualisation tool were deemed useful.

Although the majority of tasks need to be as user-friendly as possible to allow behavioural and social scientists to perform them comfortably, certain tasks, such as setting up the repository and defining the relationships between spreadsheets, need to be carried out by a project administrator with suitable technical knowledge.

## 3 Implementation

OntoSpreadEd uses the GitHub platform for its central storage. In addition, many of GitHub’s other features support the development and discussion of ontologies and their contents. For example, GitHub is designed around the version control system G *it* ^4^ allowing developers to track changes among multiple files, work simultaneously on the same files without (or with as few as possible) conflicts and manage different versions of the same repository at the same time ( *branches*). Other frequently used features include GitHub’s issue and discussion functionality, in which developers, users and external people can point out errors, ask for advice or start a discussion.

**Figure 1.**
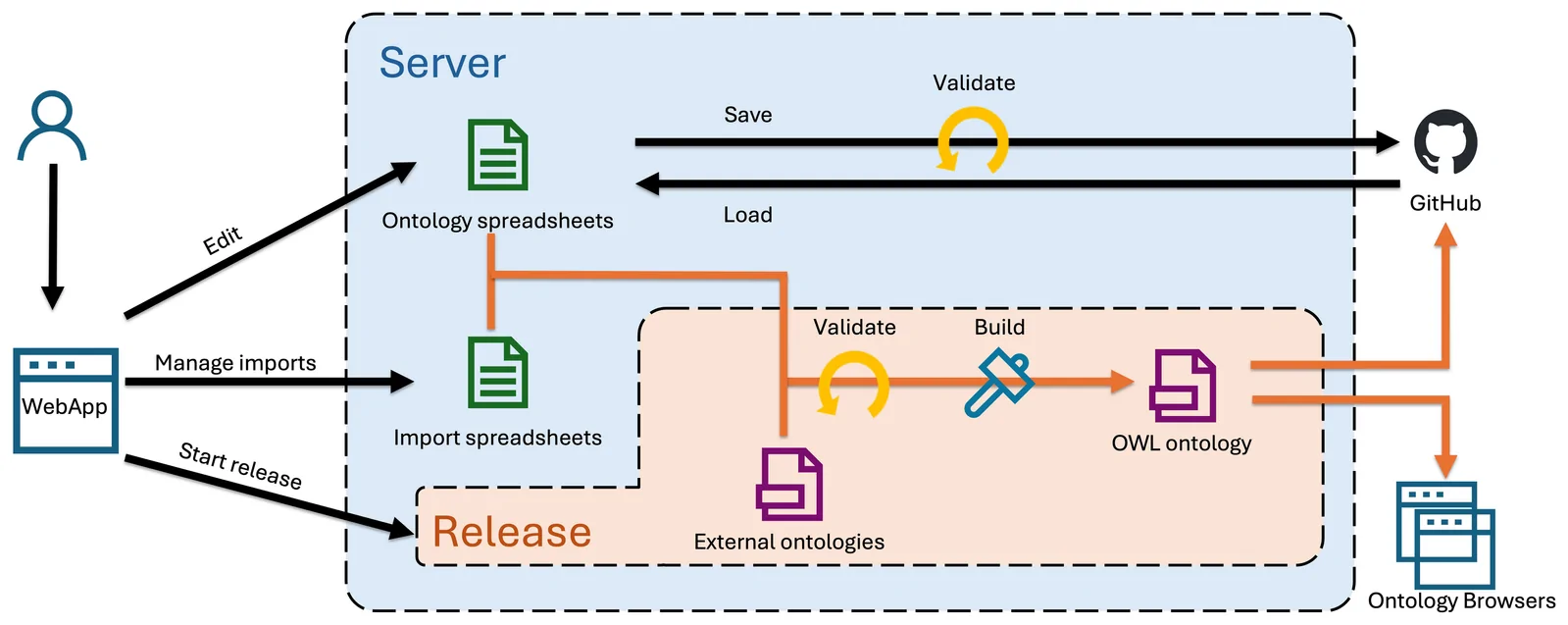
Architectural overview of OntoSpreadEd. Users view and edit the spreadsheets through a web application. When they save a file, the system validates their changes. Users can start a release process which performs a more thorough validation check, converting the spreadsheets to OWL files and publishing them.

Figure 1 shows the overall architecture of OntoSpreadEd. It is built as a web application, designed to run in a browser, based on Python and Flask. Users can interact with spreadsheets stored on GitHub through OntoSpreadEd.

The integration with GitHub requires the user to login to GitHub and authorise the app to make changes on their behalf. A successful GitHub login grants access to OntoSpreadEd ^5^. Changes made through OntoSpreadEd are sent to the GitHub repository. Because these changes may lead to *merge conflicts* (i.e. they clash with changes from another user), they are first pushed to a separate *branch* – a new version of the repository existing in parallel with the main version. A *pull request* to merge the new branch with the main branch is then created. This allows users to view the changes and inspect potential *merge conflicts*. However, if the changes do not conflict with the main version, OntoSpreadEd automatically closes the *pull request* and merges the changes with the main *branch*.

All spreadsheets, along with additional documents and materials, are stored and versioned on GitHub, enabling users to download and upload files manually (e.g., for offline work) or edit them in other programs, such as Protégé. To ensure that the spreadsheets remain usable outside of OntoSpreadEd, formatting, such as coloured rows and formatted text, must be embedded directly in the spreadsheets rather than being solely managed by OntoSpreadEd. Since plain CSV files do not support such customisations, the spreadsheets are stored in Excel format (.xlsx). For more details on the structure and function of these spreadsheets, see Section 3.1.

During the development of ontologies, reusing existing ontology classes and properties can enhance interoperability across ontologies and ensure consistency across existing and well-established ontologies ( Jackson *et al.*, 2021). Reusing ontology entities also reduces duplication, saves time and resources and supports standardisation across applications. OntoSpreadEd uses a dedicated spreadsheet to hold a list of external ontologies, from which external entities are imported. From each external ontology, it extracts the listed external entities and intermediate entities along the hierarchy.

To compile the spreadsheets to OWL ontologies, a user can start a *release*. During a *release*, OntoSpreadEd validates all spreadsheets searching for potential errors and presents errors and warnings to the user. Once no errors remain, the spreadsheets are converted to OWL using ROBOT ( Jackson *et al.*, 2019). Before publishing the built OWL files, OntoSpreadEd offers the user the possibility of manually inspecting the files and looking for anything unusual, such as unexpected labels or misplaced classes in the hierarchy. If the user finds the ontologies correct and ready to be released, the files are uploaded to GitHub, where they get picked up by other tools, including the Ontology Lookup Service (OLS ^6^) ( Côté *et al.*, 2006). In addition, OntoSpreadEd can directly update the release data in associated ontology-specific browsers, including BCIOsearch ^7^. Section 3.3 further explains the release process.

### 3.1 Spreadsheet format and schema

**Figure 2.**
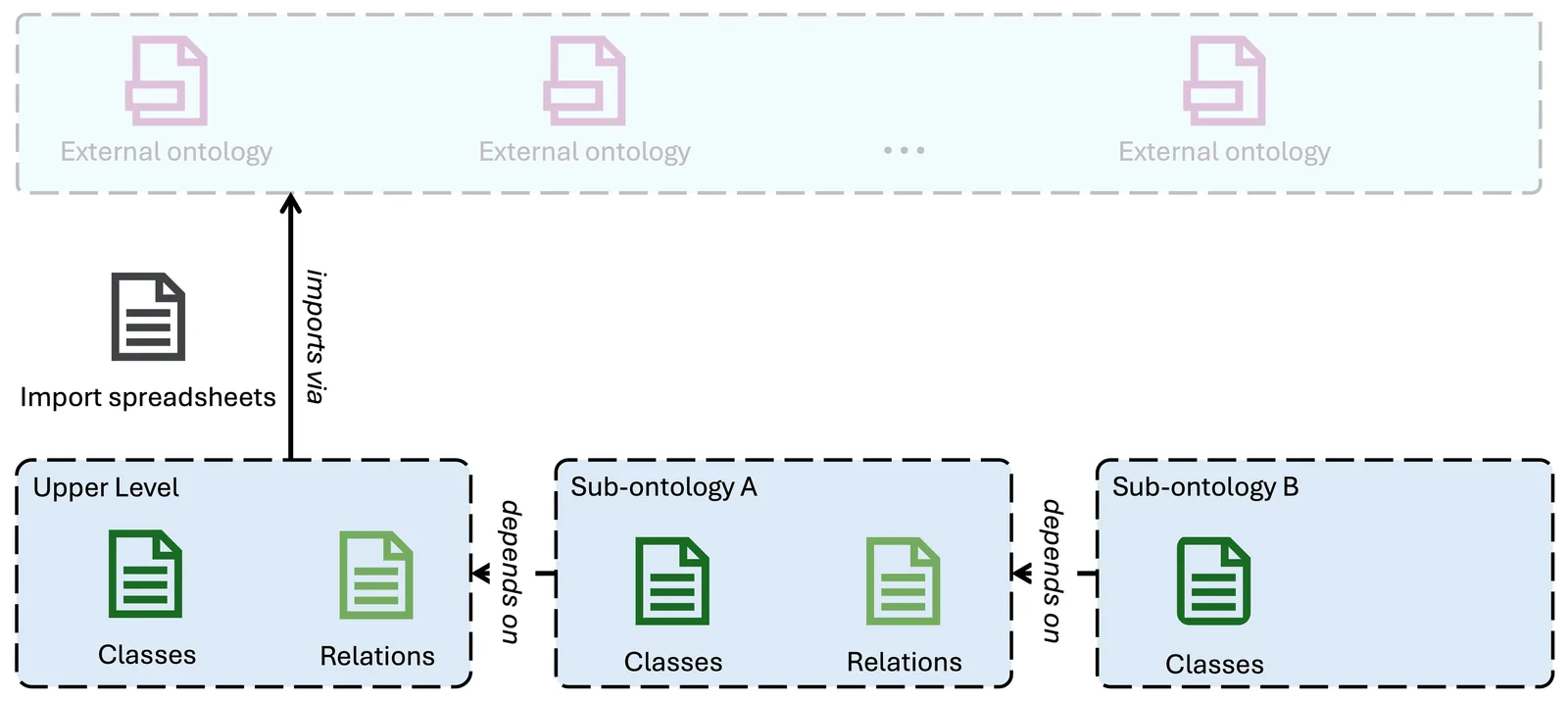
The three different kinds of spreadsheets OntoSpreadEd works with. An ontology managed by OntoSpreadEd consists of spreadsheets storing (1) classes, (2) relations and (3) a list of external ontologies and terms to import. Each class (and relations) spreadsheet can form a subontology that can depend on other subontologies and be released in separation.

There are three different types of spreadsheets with which OntoSpreadEd works: Spreadsheets defining classes ( *class spreadsheet*) and properties ( *relations spreadsheet*), as well as one that manages imports from external ontologies ( *importing spreadsheet*). Figure 2 depicts how each is used to compose the ontology.

Each row in a *class spreadsheet* defines an (OWL) class, which is uniquely identified by its ID. In addition, a class’s label must be unique, allowing users to refer to a class via its label instead of its ID. OntoSpreadEd auto-generates IDs for new classes. Similarly, each row in a relations spreadsheet defines a *Relation* (mapped to an OWL property) with an ID and label. There may be multiple classes or relations spreadsheets for a single ontology.

To import from external ontologies, there is a dedicated *importing spreadsheet* in the repository. Each row in the *importing spreadsheet* contains information about the external ontology and lists the entities to be imported.

The *class spreadsheets* and *relation spreadsheets* are converted to OWL during a *release*. The mapping of spreadsheet columns to OWL follows a customisable schema. By default, the following mappings are used:

- The **ID** column is mapped to the IRI of the class or relation.
- The **Label** column is mapped to rdfs:label.
- The **Parent** column is mapped to SubClassOf.
- The **Logical definition** column contains a logical, formal expression of the class mapped to an OWL EquivalentClasses axiom. The class expression must be written in the OWL Manchester Syntax ( Horridge *et al.*, n.d.), a human-readable syntax for the OWL language.
- The **Disjoint classes** column explicitly lists other classes which are distinct from the class represented by the row; it is mapped to an OWL DisjointClasses axiom.
- Columns about relations to other classes follow the format “REL *X*” where *X* is the label of an object property. Such a relation is represented in OWL as an axiom of the form SubClassOf some X.

Table 1 shows an example of a *class spreadsheet:* each row represents a class and each column illustrates one of the above-mentioned cases. Here, the first columns define the ID, label and parent of each class. Additionally, the class “human behaviour” is enriched with a logical definition; the "behaviour change intervention” class is set in a “has process part” relation with “behaviour change intervention outcome behaviour”; and the “pull mode of delivery” class is explicitly marked as distinct from the “push mode of delivery”.

**Table 1.** Example illustrating a class spreadsheet with three entities. An example illustrating a class spreadsheet. It contains three different entities where the mandatory fields “ID”, “Label” and “Parent” are set for all three entities. Three additional, optional columns illustrate other kinds of fields.

ID	Label	Parent	Logical definition	Disjoint classes	REL 'has process part'
BCIO:042000	human behaviour	process	"individual human behaviour" OR "population behaviour"		
BCIO:003000	behaviour change intervention	intervention			behaviour change intervention outcome behaviour
BCIO:011063	pull mode of delivery	behaviour change intervention mode of delivery		push mode of delivery	

**Figure 3.**
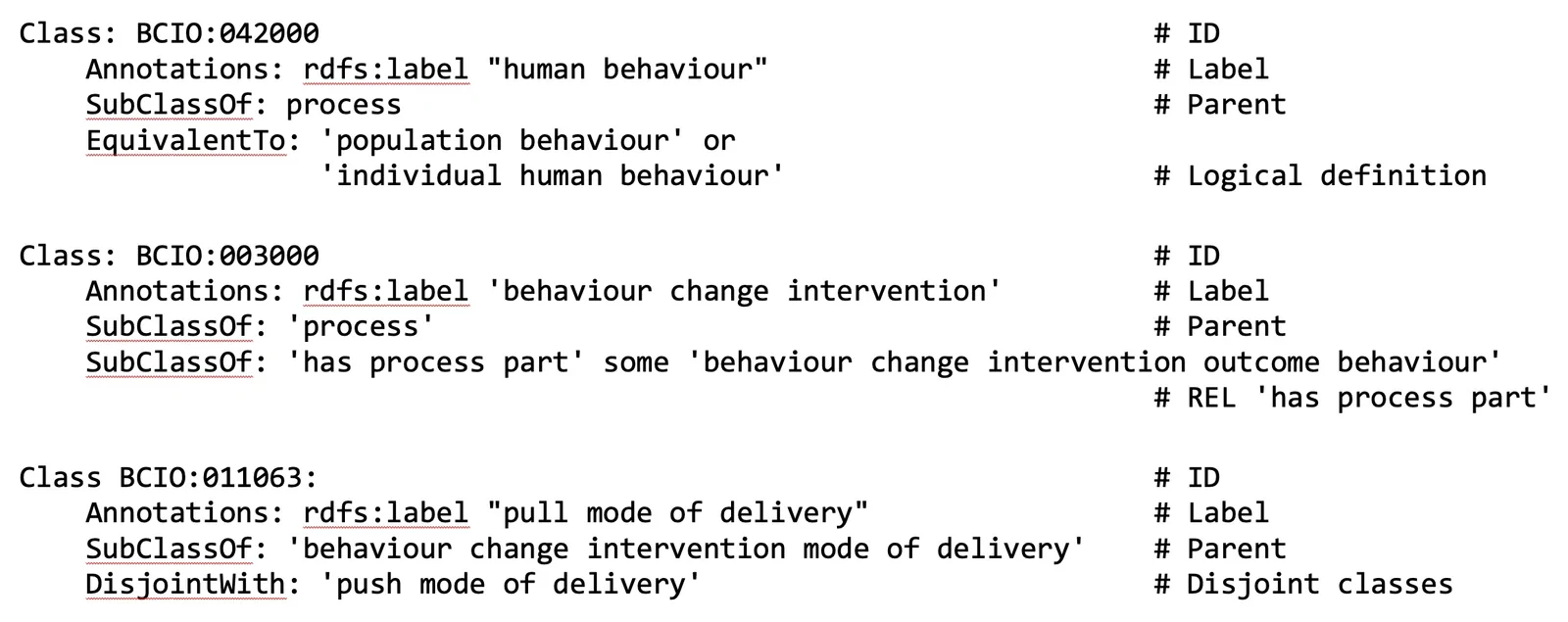
Resulting OWL from a spreadsheet as illustrated in Table 1. Note, that for better readability, labels have not been replaced by IDs in this example. Each row from the spreadsheet in Table 1 is transformed to an OWL structure.

The table illustrating an exemplary spreadsheet in Table 1 results in an OWL ontology, as shown in Figure 3.

Additionally, the schema defines column mappings for certain, often-used relations, which are mapped to annotations (OWL AnnotationAssertions), for example, *Comment* (mapped to rdfs:comment), *Synonyms* (mapped to IAO:0000118 ) or *Curation status* (mapped to IAO:0000114 ). Additionally, columns can be marked as internal notes or flagged to be completely ignored by the system; in both cases, such columns are not published and are not included in the published OWL files. Internal columns can be used for reviewing remarks or additional information needed, for example, for the integration with ontology browsers.

### 3.2 Enhancements over generic spreadsheet editors

The core interface of OntoSpreadEd is very similar to a plain spreadsheet editor such as Excel or LibreOffice Calc. However, OntoSpreadEd offers important usability features to enhance the workflow for ontology development.

***Auto filling fields***

Some fields are automatically filled or updated by OntoSpreadEd. For example, the “Curator” field is updated, when a user changes a row, or the curation status is set to “External” when the ID of an entity does not belong to the current ontology.

***Autocomplete***

In fields which expect entities or only allow for a selected list of options, OntoSpreadEd suggests valid options or auto-completes the user’s input.

***Validation***

The ROBOT tool only signals possible errors when converting spreadsheets to OWL, typically only one by one, and not always in a way that is understandable for most users. OntoSpreadEd validates the spreadsheets manually, both on save and during release, trying to find potential errors beforehand. Section 3.4 provides more details about validation.

***Searching***

OntoSpreadEd maintains a search index to allow users to search for classes across spreadsheets. Among the search functionality, it also powers the autocomplete suggestions.

***Visualisation***

Tools have been developed to visualise the hierarchy of OWL ontologies. In addition, OntoSpreadEd allows the user to visualise a spreadsheet or even a selection of rows in the context of the rest of the ontology to view the effects of changes, even before saving.

***Resolving merge conflicts***

Version control systems, such as Git, are by default unable to merge Excel spreadsheets, leading to the added work of manual conflict resolution. OntoSpreadEd can detect conflicts while merging, automatically merge unconflicting changes and assist in resolving conflicts.

### 3.3 Conversion to OWL (Release)

The core of the release of an ontology via OntoSpreadEd is the conversion to OWL. However, the spreadsheet and file structure differ from ontology to ontology. In particular, an ontology may consist of multiple lower-level ontologies which should be released independently as well, or it might consist of only one target release as a single file. For example, the setup depicted in Figure 2 shows an ontology containing an “Upper Level” ontology and two lower-level ontologies “A” and “B”. Here, “A” builds on top of “Upper Level” and “B” builds on top of “A”. This hierarchy of ontologies enables the release of “A” independently of “B” and ensures that all classes and relations from “A” and “Upper Level” are included (and accessible) from “B”.

These kind of dependencies and other details, such as the name of the ontology, must be defined in a *release configuration*. The *release configuration* also contains the list of source spreadsheets which correspond to each OWL file to be built and defines which steps should be executed and with which parameters (e.g. the files for which a hierarchical spreadsheet should be generated).

A release undergoes several phases:

**Preparation**

During this first step, all spreadsheets relevant to the release are downloaded to the server. All further steps will work on this snapshot to prevent changes made during a release (e.g. by another person) from interfering with the process.

**Building external ontologies**

Each external ontology listed in the corresponding spreadsheet is downloaded to the server. ROBOT is then used to extract a subset of the ontologies containing the entities to import using ROBOT’s MIREOT ( Courtot *et al.*, 2011) strategy. Finally, all resulting subsets are merged into one. The process is optimized with caches to only download and extract ontologies on changes. This step can also be configured to run automatically between releases to ensure auto-complete and validation know about newly imported entities.

**Validation**

This phase checks for (potential) errors and warnings in spreadsheets (see Section 3.4 for more details). If OntoSpreadEd identifies (potential) problems, they are presented to the user with a link to the origin. Some of these issues can be attempted to be resolved automatically. The release can only continue if no errors are found.

**Building the ontology**

According to the schema (see Section 3.1), OntoSpreadEd converts the spreadsheets to OWL files using ROBOT and ROBOT templates.

**Merging files**

If necessary, the previously built OWL files are merged into a single releasable file. Additionally, OWL imports are resolved to produce a single, self-contained ontology file.

**Human verification**

OntoSpreadEd presents the built files to the user before publishing them. The user is asked to verify the OWL files by inspecting them (for example, in Protégé).

**Updating ontology browsers**

If configured, OntoSpreadEd updates ontology browsers with the new data.

**Hierarchical spreadsheets**

If configured, OntoSpreadEd builds simplified versions of the spreadsheets, reflecting the hierarchy by indenting children.

**Upload to GitHub**

As a final step, the built OWL files are uploaded to GitHub, from where they can be picked up by other tools such as OLS.

### 3.4 Internal representation and validation

Each class or relation is uniquely identified by its ID. OWL only requires IRIs (i.e. a browsable address on the Internet; see Section 1.1) as unique identifiers. Fortunately, an ID is just a CURIE (i.e. an abbreviated IRI; see Section 1.1) and can thus be used directly in OWL. In accordance with OBO principles ( Jackson *et al.*, 2021), OntoSpreadEd requires labels to be unique as well. This allows for a 1:1 mapping of IDs to labels, enabling users to refer to other entities, for example their parent, by their label instead of their ID.

Common mistakes when developing an ontology are simple typos when writing the label of a referenced entity, renaming an entity but not adjusting its usage and deleting an entity or declaring it obsolete while it is still referred to. Another typical error occurs when an entity is used, for example, as a parent, but it is declared in another spreadsheet or even an external ontology and not imported (properly). These mistakes make it impossible to match labels to their respective IDs. During validation, OntoSpreadEd reports such occurrences as *errors*. Other checks verify that mandatory fields (ID, Label and Definition) are set and that external entities are properly imported and integrated. In addition, the system checks for conflicting duplicate entries across all spreadsheets.

In addition to *errors*, which prevent OntoSpreadEd from translating the spreadsheets to OWL, the system issues *warnings* if a spreadsheet or row is technically correct but may lead to unintended results. For example, if an external entity is imported and also listed in a spreadsheet but some of its relations or annotations (e.g. its definition) are different, it can still be translated to OWL but will result in two different definitions.

### 3.5 Operation

The following provides an overview of the software and hardware requirements for a system to run an instance of OntoSpreadEd.

OntoSpreadEd requires Python and depends on several Python packages. Among the most important ones are *Flask*, powering the webserver, *openpyxl*, allowing Excel files to be processed in Python, as well as *py-horned-owl* ( Lord *et al.*, 2024) and *ROBOT* ( Jackson *et al.*, 2019) used to process OWL files.

A GitHub OAuth app is required for GitHub integration.

As a web application, OntoSpreadEd does not have notable minimum hardware requirements. The only exception is the release, where external ontologies are downloaded and loaded to RAM during processing. Hence, the running server must have sufficient memory available to load the largest imported ontology.

To integrate a repository into OntoSpreadEd, it is expected to have a configuration file .onto-spread-ed/config.yaml. The administrator can configure a list of repositories to be loaded upon startup. Additionally, users can load further repositories (if allowed by the administrator). Users must have read and write access to the repositories they want to edit using OntoSpreadEd.

OntoSpreadEd enforces an *allowlist* permitting access to only a configured list of users to protect the instance against abuse.

## 4 Discussion and Conclusions

OntoSpreadEd is an accessible, user-friendly tool that enables collaboration between domain experts and technical experts in developing, updating and reviewing ontologies. It uses a familiar spreadsheet interface and then uses ROBOT ( Jackson *et al.*, 2019) to transform the spreadsheets to OWL files. Added automated validation, both for each spreadsheet and before the release of the ontology, reduces the risk of human error throughout development. OntoSpreadEd integrates with GitHub as a version control system and with ontology browsers, such as the Ontology Lookup Service and, if configured, others such as BCIOSearch.

OntoSpreadEd is a step towards broader adoption of ontologies and ontology tools within the behavioural and social sciences, allowing for collaboration between people from different disciplines with a range of different requirements and technical expertise.

### 4.1 Limitations and directions

There are a number of limitations still remaining in the current version of OntoSpreadEd.

First, while using OntoSpreadEd does not require a technical background, setting it up still requires a server and some configuration that goes beyond the expertise of most researchers in the behavioural and social sciences. While some universities provide technical support for these kinds of tasks, this is unfortunately not always the case.

Second, OntoSpreadEd relies on its integration with GitHub. There are some downsides of GitHub; most notably, it is a proprietary platform owned by Microsoft. Alternatives that correspond more closely to the values of open infrastructure (e.g. Codeberg, GitLab) are more desirable for projects that highly value open science principles but are less established and less well-supported by existing packages. Similarly, OntoSpreadEd uses .xlsx format for the spreadsheets. This is a proprietary format, created by Microsoft. However, while open-tabular formats exist, most commonly .csv files, they do not offer all the functionalities of.xlsx files. Most notably, .xlsx files were chosen to be able to use colours and formatted text within the tables, making it easier to navigate and thus more accessible.

Third, the expressiveness in OntoSpreadEd is limited; not everything expressible with OWL can be expressed with OntoSpreadEd at the moment. For example, relations can only be existential (e.g. “a study has *some* adult participants”) but not universal (e.g. “a study has *only* adult participants”) and working with OWL individuals is currently generally not supported. We plan to incorporate these features into OntoSpreadEd in the future.

Fourth, at this point, documentation of OntoSpreadEd is relatively limited, with internal users receiving online training. We are currently working on a more elaborate user guide, as well as additional resources, such as tutorial videos, to support external users of OntoSpreadEd.

Although OntoSpreadEd has been used by a range of users with a background in behavioural and social sciences, its usability has not been systematically tested with external users. In the future, we plan to formally test the usability of OntoSpreadEd, potentially in comparison with other popular ontology editors, with a range of users. This can be done quantitatively, for example, by exploring the speed at which different tasks can be carried out in different editors ( Musen & Protégé Team, 2015), or qualitatively, for example, using think-aloud studies.

## Glossary of terms

***Ontology:***

A machine-readable way of representing knowledge, which usually includes unique identifiers, labels, and definitions for each thing that exists within a domain, as well as well-defined relationships between them ( Arp *et al.*, 2015).

***OWL*:**

Web Ontology Language; a common language used for ontology development

***Entity*:**

Anything that can be defined in OWL is termed an entity and; includesing classes, object-, data,- and data properties.

***IRI:***

Internationalized Resource Identifier:; Aa browsable address on the Iinternet with support for a wide range of characters, including those from different languages.

***CURIE:***

Compact URI; an abbreviation for an *IRI*. For example, the IRI http://purl.obolibrary.org/obo/BFO_0000001 can be shrunk to the CURIE BFO:0000001 (assuming a prefix BFO is defined as http://purl.obolibrary.org/obo/BFO_).

***Class:***

OWL component representing a thing that exists in the universe.

***Sub-class* **and** *Super-class:***

The class representing something more general is the *super class* of the class representing something more specific ( *sub-class*).

***Object properties:***

Relations connecting two OWL classes

***Data properties:***

Relations connecting an OWL class and plain data (e.g., numbers, text, and dates)

***Annotation properties:***

Relations for adding textual (non-formal) information to entities

***Git:***

A version control system keeping track of changes from different users over time

***GitHub*:**

Platform for hosting repositories managed via git

***Branch:***

Another version of thea git- managed repositor. existing in parallel with the other versions, and the main version

***Merge conflict:***

Conflicts arising when clashing changes from different people are tried to be merged

***Pull request:***

TheA concept of GitHub to merge two different branches with possibilities to review and comment on the changes.

***Release:***

A release with OntoSpreadEd converts the ontology developed in the spreadsheets into distributable OWL files.

***Release configuration:***

A configuration file specifying details about the setup of spreadsheets

***Class spreadsheets:***

Spreadsheets containing definitions of one class per row

***Relation spreadsheets:***

Spreadsheets containing definitions of one object-, data- and annotationproperties per row

***Importing spreadsheet:***

One special spreadsheet per ontology which lists external ontologies and entities to import from them

***Allowlist:***

A list with allowed users; only users on that list can access OntoSpreadEd

## Ethical considerations

Ethical approval and consent were not required.

## Data Availability

No data associated with this article.

## Footnotes

^1^ https://github.com

^2^ https://gitlab.com

^3^ For more information on existing ontology editors, see ( Cardoso & Sheth, 2006; Mendonça *et al.*, 2021)

^4^ https://git-scm.com/

^5^ To avoid abuse OntoSpreadEd instances additionally configure an allowlist of users

^6^ https://www.ebi.ac.uk/ols4/

^7^ https://bciosearch.org

## Software availability statement

Source code available from: https://github.com/ontology-tools/onto-spread-ed/

Archived software available from: https://doi.org/10.5281/zenodo.15673812

License: GNU Lesser General Public License v3.0 only
