# Supplementary material for: OntoSpreadEd: Developing Ontologies with a Dedicated Online Template Spreadsheet Editor
Source: Wellcome Open Res. 2026 Jul 5;10:360. Originally published 2025 Jul 25. [Version 2] doi: 10.12688/wellcomeopenres.24408.2 (PMC13550900; doi:10.12688/wellcomeopenres.24408.2)
Supplement: Peer review history

# Reviewer report: Reviewer response for version 2

**DOI:** 10.21956/wellcomeopenres.28988.r159928

**Published:** 2026-07-11

**Authors:** Ray Stefancsik

**Affiliations:** 1 European Bioinformatics Institute (EMBL-EBI), Cambridgeshire, UK

The revised version appears to be an improvement compared with the earlier one. The paper looks fine overall.

 An area for improvement would be to define all abbreviations upon first use, to benefit readers who may not be familiar with them (e.g., OBI, OBA in paragraph 5 of section "1.2 Tools to develop and evaluate ontologies").

Are the conclusions about the tool and its performance adequately supported by the findings presented in the article?

Yes

Is the rationale for developing the new software tool clearly explained?

Yes

Is the description of the software tool technically sound?

Yes

Are sufficient details of the code, methods and analysis (if applicable) provided to allow replication of the software development and its use by others?

Yes

Is sufficient information provided to allow interpretation of the expected output datasets and any results generated using the tool?

Yes

Reviewer Expertise:

biomedical ontologies; biomedical data; genetics; molecular biology

I confirm that I have read this submission and believe that I have an appropriate level of expertise to confirm that it is of an acceptable scientific standard.

# Reviewer report: Reviewer response for version 2

**DOI:** 10.21956/wellcomeopenres.28988.r159927

**Published:** 2026-07-10

**Authors:** Arkalgud Ramaprasad

**Affiliations:** 1 University of Illinois at Chicago, Chicago, USA

The authors have addressed my comments well. Thank you.

Are the conclusions about the tool and its performance adequately supported by the findings presented in the article?

Partly

Is the rationale for developing the new software tool clearly explained?

Yes

Is the description of the software tool technically sound?

Yes

Are sufficient details of the code, methods and analysis (if applicable) provided to allow replication of the software development and its use by others?

Yes

Is sufficient information provided to allow interpretation of the expected output datasets and any results generated using the tool?

Yes

Reviewer Expertise:

Information systems and decision sciences

I confirm that I have read this submission and believe that I have an appropriate level of expertise to confirm that it is of an acceptable scientific standard.

# Reviewer report: Reviewer response for version 1

**DOI:** 10.21956/wellcomeopenres.26912.r128684

**Published:** 2025-09-11

**Authors:** Arkalgud Ramaprasad

**Affiliations:** 1 University of Illinois at Chicago, Chicago, USA

The paper is focused on a tool to aid the development and use of OWL-based ontologies. The tool seeks to address the challenges arising from the complexity of these ontologies and the use of OWL and OWL-related tools to manage them. As a tool with this limited focus, it appears to be effective, albeit with the limitations mentioned in the paper. The fact that it is spreadsheet-based makes it accessible to more users and easy to use. However, the paper overreaches in many of its statements and assertions. I shall highlight and comment on them in the following. I have not included citations in the following.

 They are available in papers (co)authored by me, many of which are in the public domain and easily located since this is an open review.  

 “Ontologies are machine-readable representations of knowledge that can accelerate research across various disciplines.”

 Ontologies encapsulate the logic of being, as opposed to epistemologies that encapsulate the logic of knowing. Some encapsulate the terminologies and taxonomies of a domain. They may not necessarily be in a machine-readable form, unless one considers any form of optical character recognition as being machine readable. Ontologies can be machine-readable representations …. OWL-based ontologies are a type of ontology. There can be other types. The authors may want to investigate.

 “To build ontologies, domain scientists must formally capture their knowledge of their domains in structured forms.”

 True, but only partly. Ontologies can capture morphological and syntactic knowledge well, but the semantic and pragmatic knowledge only to a limited extent.  

 “The resulting technically oriented tools create barriers to adoption and use by nontechnical domain experts.”

 Could ontologies, accessible to both novices and experts, be constructed without complex tools? After all, ontologies are simply organization of words, the terminologies and taxonomies of the domain.

 “The Gene Ontology has been cited more than 40,000 times, demonstrating its immense influence in the field of biology.”

 “In recent years, ontologies have started to be adopted within the behavioural and social sciences (Hastings *et al.*, 2024).”

 “Ontologies are not yet widely used in the behavioural and social sciences due in part to a lack of tools to support their development and maintenance.”

 True. But OWL-based ontologies are particularly suited for domains with a structured terminology and that have an ongoing effort to maintain the terminology. Not many domains in the social sciences have a similar structured terminology and a constant effort to maintain it. The very limited examples of adoption of OWL-based ontologies may be as much due to the inapplicability of such ontologies to the domains as to the difficulty of using the tool.

 “Ontologies are typically developed in OWL …”

 Not necessarily, only OWL-based ontologies.

 “Tools for ontology development and editing can be challenging to use for those that are unfamiliar with them and have interfaces that tend to be unintuitive (Mendonça *et al.*, 2021). We argue that this is especially the case for users in the behavioural and social sciences, where researchers do not frequently need to use these kinds of tools and therefore do not have the experience to help them learn to use the tools.”

 True if the only types of ontologies are OWL-based. The authors may want to take a broader perspective. Many ontologies can be simply sketched by hand or constructed directly on a spreadsheet or word processor.

 “Additionally, the class “human behaviour” is enriched with a logical definition; the “behaviour change intervention” class is set in a “has process part” relation with “behaviour change intervention outcome behaviour”; the “pull mode of delivery” class is explicitly marked as distinct from the “push mode of delivery”.

 The challenge of applying OWL-type ontologies is illustrated by the above definitions and the logical definition of human behaviour as “individual human behaviour” OR “population behaviour” in Table 1. The dichotomous definition of human behaviour is simplistic, incomplete, and inadequate. There are many – more elaborate and comprehensive taxonomies – of human behaviour are available in the literature. It may be appropriate to import one suited for the context at hand and adapt it. The same comment applies to the push-pull dichotomy.

 In conclusion, the tool may be appropriate for domains with well-structured and curated terminologies. It is unlikely to extend the application of OWL-based ontologies to the behavioural and social sciences. The latter need a different approach.

 The paper is focused on a tool to aid the development and use of OWL-based ontologies. The tool seeks to address the challenges arising from the complexity of these ontologies and the use of OWL and OWL-related tools to manage them. As a tool with this limited focus, it appears to be effective, albeit with the limitations mentioned in the paper. The fact that it is spreadsheet-based makes it accessible to more users and easy to use. However, the paper overreaches in many of its statements and assertions. I shall highlight and comment on them in the following. I have not included citations in the following. They are available in papers (co)authored by me, many of which are in the public domain and easily located since this is an open review.  

 “Ontologies are machine-readable representations of knowledge that can accelerate research across various disciplines.”

 Ontologies encapsulate the logic of being, as opposed to epistemologies that encapsulate the logic of knowing. Some encapsulate the terminologies and taxonomies of a domain. They may not necessarily be in a machine-readable form, unless one considers any form of optical character recognition as being machine readable. Ontologies can be machine-readable representations …. OWL-based ontologies are a type of ontology. There can be other types. The authors may want to investigate.

 “To build ontologies, domain scientists must formally capture their knowledge of their domains in structured forms.”

 True, but only partly. Ontologies can capture morphological and syntactic knowledge well, but the semantic and pragmatic knowledge only to a limited extent.  

 “The resulting technically oriented tools create barriers to adoption and use by nontechnical domain experts.”

 Could ontologies, accessible to both novices and experts, be constructed without complex tools? After all, ontologies are simply organization of words, the terminologies and taxonomies of the domain.

 “The Gene Ontology has been cited more than 40,000 times, demonstrating its immense influence in the field of biology.”

 “In recent years, ontologies have started to be adopted within the behavioural and social sciences (Hastings *et al.*, 2024).”

 “Ontologies are not yet widely used in the behavioural and social sciences due in part to a lack of tools to support their development and maintenance.”

 True. But OWL-based ontologies are particularly suited for domains with a structured terminology and that have an ongoing effort to maintain the terminology. Not many domains in the social sciences have a similar structured terminology and a constant effort to maintain it. The very limited examples of adoption of OWL-based ontologies may be as much due to the inapplicability of such ontologies to the domains as to the difficulty of using the tool.

 “Ontologies are typically developed in OWL …”

 Not necessarily, only OWL-based ontologies.

 “Tools for ontology development and editing can be challenging to use for those that are unfamiliar with them and have interfaces that tend to be unintuitive (Mendonça *et al.*, 2021). We argue that this is especially the case for users in the behavioural and social sciences, where researchers do not frequently need to use these kinds of tools and therefore do not have the experience to help them learn to use the tools.”

 True if the only types of ontologies are OWL-based. The authors may want to take a broader perspective. Many ontologies can be simply sketched by hand or constructed directly on a spreadsheet or word processor.

 “Additionally, the class “human behaviour” is enriched with a logical definition; the “behaviour change intervention” class is set in a “has process part” relation with “behaviour change intervention outcome behaviour”; the “pull mode of delivery” class is explicitly marked as distinct from the “push mode of delivery”.

 The challenge of applying OWL-type ontologies is illustrated by the above definitions and the logical definition of human behaviour as “individual human behaviour” OR “population behaviour” in Table 1. The dichotomous definition of human behaviour is simplistic, incomplete, and inadequate. There are many – more elaborate and comprehensive taxonomies – of human behaviour are available in the literature. It may be appropriate to import one suited for the context at hand and adapt it. The same comment applies to the push-pull dichotomy.

 In conclusion, the tool may be appropriate for domains with well-structured and curated terminologies. It is unlikely to extend the application of OWL-based ontologies to the behavioural and social sciences. The latter need a different approach.

Are the conclusions about the tool and its performance adequately supported by the findings presented in the article?

Partly

Is the rationale for developing the new software tool clearly explained?

Yes

Is the description of the software tool technically sound?

Yes

Are sufficient details of the code, methods and analysis (if applicable) provided to allow replication of the software development and its use by others?

Yes

Is sufficient information provided to allow interpretation of the expected output datasets and any results generated using the tool?

Yes

Reviewer Expertise:

Information systems and decision sciences

I confirm that I have read this submission and believe that I have an appropriate level of expertise to confirm that it is of an acceptable scientific standard, however I have significant reservations, as outlined above.

## References

[1] : Social Inclusion/Exclusion in Information Systems: A Review and Roadmap for Research. *Information Systems Journal* .2025;35(5) : 10.1111/isj.12582 1311-1343 10.1111/isj.12582

[2] : A systematic study of construction and demolition waste management. *Environment, Development and Sustainability* .2025; 10.1007/s10668-025-06621-6 10.1007/s10668-025-06621-6

[3] : Access to Healthcare during COVID-19. *International Journal of Environmental Research and Public Health* .2021;18(6) : 10.3390/ijerph18062980 10.3390/ijerph18062980 PMC799934633799417

[4] : Megaprojects – symbolic and sublime: an ontological review. *International Journal of Managing Projects in Business* .2019;12(2) : 10.1108/IJMPB-03-2018-0054 377-399 10.1108/IJMPB-03-2018-0054

# Response

**Published:** 2026-07-06

**Authors:** Björn Gehrke

**Affiliations:** Idiap Research Institute, Martigny, Valais, Switzerland

We want to thank you for the constructive feedback and comments on our manuscript. Your contribution has greatly helped us make the manuscript not only clearer but also more contextualised. Below, please find a point-by-point reply to the comments we have received. *Ontologies encapsulate the logic of being, as opposed to epistemologies that encapsulate the logic of knowing. Some encapsulate the terminologies and taxonomies of a domain. They may not necessarily be in a machine-readable form, unless one considers any form of optical character recognition as being machine readable. Ontologies can be machine-readable representations …. OWL-based ontologies are a type of ontology. There can be other types. The authors may want to investigate.*

 Thank you for this important comment. We agree that not everything that can be considered an ontology must be machine-readable and OWL-based. We changed the text of our introduction to clarify that we are describing the scope of the current paper. *Could ontologies, accessible to both novices and experts, be constructed without complex tools? After all, ontologies are simply organization of words, the terminologies and taxonomies of the domain.*

 Thank you for this comment. We agree that this would be ideal. The tool tries to be a step in the direction.

*“Ontologies are typically developed in OWL …” Not necessarily, only OWL-based ontologies.*

 Thank you for this comment. We agree that not everything that can be considered an ontology must be OWL-based. We changed the text of our introduction to clarify that we are describing the scope of the current paper.**

*“Tools for ontology development and editing can be challenging to use for those that are unfamiliar with them and have interfaces that tend to be unintuitive (Mendonça et al., 2021). We argue that this is especially the case for users in the behavioural and social sciences, where researchers do not frequently need to use these kinds of tools and therefore do not have the experience to help them learn to use the tools.”  True if the only types of ontologies are OWL-based. The authors may want to take a broader perspective. Many ontologies can be simply sketched by hand or constructed directly on a spreadsheet or word processor.* Thank you for this comment. We agree that not everything that can be considered an ontology must be OWL-based. We changed the text of our introduction to clarify that we are describing the scope of the current paper. *“To build ontologies, domain scientists must formally capture their knowledge of their domains in structured forms.” True, but only partly. Ontologies can capture morphological and syntactic knowledge well, but the semantic and pragmatic knowledge only to a limited extent.*

 Thank you for this comment. We do not believe we implied that ontologies can capture semantic and pragmatic knowledge to a full extent in the quoted sentence.**

*“The Gene Ontology has been cited more than 40,000 times, demonstrating its immense influence in the field of biology.”; “In recent years, ontologies have started to be adopted within the behavioural and social sciences (Hastings et al., 2024).”; “Ontologies are not yet widely used in the behavioural and social sciences due in part to a lack of tools to support their development and maintenance.”  True. But OWL-based ontologies are particularly suited for domains with a structured terminology and that have an ongoing effort to maintain the terminology. Not many domains in the social sciences have a similar structured terminology and a constant effort to maintain it. The very limited examples of adoption of OWL-based ontologies may be as much due to the inapplicability of such ontologies to the domains as to the difficulty of using the tool.*

*In conclusion, the tool may be appropriate for domains with well-structured and curated terminologies. It is unlikely to extend the application of OWL-based ontologies to the behavioural and social sciences. The latter need a different approach.*

 Thank you for your comment. We agree that behavioural and social sciences have historically been viewed as more complex to formalize than some natural sciences. However, given the existence and widespread use of many taxonomies and classification systems with weaker semantic specification (e.g. diagnostic manuals, behaviour change technique taxonomy, compendium of physical activities, …), we believe that there is in fact great potential for ontologies in behavioural sciences. This is also supported by the consensus report by the national academy of sciences (https://nap.nationalacademies.org/catalog/26464/ontologies-in-the-behavioral-sciences-accelerating-research-and-the-spread) calling specifically for formal ontologies to accelerate behavioural science, and by the growing membership of associated communities of practice such as the BSSO Foundry (https://wellcomeopenresearch.org/articles/9-656/v1) and the APRICOT Community of Practice.**

*“Additionally, the class “human behaviour” is enriched with a logical definition; the “behaviour change intervention” class is set in a “has process part” relation with “behaviour change intervention outcome behaviour”; the “pull mode of delivery” class is explicitly marked as distinct from the “push mode of delivery”.  The challenge of applying OWL-type ontologies is illustrated by the above definitions and the logical definition of human behaviour as “individual human behaviour” OR “population behaviour” in Table 1. The dichotomous definition of human behaviour is simplistic, incomplete, and inadequate. There are many – more elaborate and comprehensive taxonomies – of human behaviour are available in the literature. It may be appropriate to import one suited for the context at hand and adapt it. The same comment applies to the push-pull dichotomy.*

 Thank you for this comment. We want to highlight that the content of the definitions is not part of the work being presented in this paper, and rather an example from a formal ontology within behavioural sciences on how OWL definitions could work. If the reviewer would like to engage further regarding specific definitions in the BCIO, we would like to encourage them to do so using the BCIO issue tracker (https://github.com/HumanBehaviourChangeProject/ontologies/issues).

 Since the original submission of this manuscript, the class “human behaviour” has been obsoleted within the BCIO based on the issue https://github.com/HumanBehaviourChangeProject/ontologies/issues/1126. As such, we have decided to replace the example in the manuscript with “coping behaviour”.

# Reviewer report: Reviewer response for version 1

**DOI:** 10.21956/wellcomeopenres.26912.r128686

**Published:** 2025-08-30

**Authors:** Ray Stefancsik

**Affiliations:** 1 European Bioinformatics Institute (EMBL-EBI), Cambridgeshire, UK

This paper presents OntoSpreadEd, a web-based spreadsheet-like editor designed to make ontology development more accessible to domain experts who lack technical training in OWL and formal ontology editing tools. The work addresses a significant barrier in the field: while ontologies can accelerate research across disciplines, existing editing tools typically require specialized knowledge of OWL and technical interfaces, limiting their adoption by domain scientists.

 * Strengths

   * Clear problem identification: the authors effectively articulate the gap between the potential benefits of ontologies and their limited adoption due to technical barriers. The motivation for developing a more accessible tool is well-established.

   * User-centered design: OntoSpreadEd's spreadsheet interface leverages familiar mental models, potentially lowering the cognitive load for domain experts transitioning to ontology development.

   * Comprehensive feature set: The tool includes essential usability features such as autocomplete, validation, and search functionality. The validation system appears particularly valuable, addressing common user errors including typos in entity references, inconsistent entity usage, missing imports, unfilled mandatory fields, and conflicting duplicate entries.

   * Integration with Version Control: The GitHub integration for version control demonstrates awareness of collaborative development needs in research environments.

   * Comparative analysis: The discussion of existing tools (Protégé, WebProtégé, onto4all, and ROBOT) provides useful context and clearly positions OntoSpreadEd within the current tool landscape.

   * Open Source availability: The availability of source code on GitHub supports reproducibility and community adoption.

 * Areas for Improvement

   * Incomplete tool coverage in the comparative analysis of the existing tools: While the comparison with existing tools is valuable, the paper would benefit from discussing additional relevant approaches:

      * Dead Simple OWL Design Pattern (DOS-DP) exchange format (Osumi-Sutherland et al., 2017, DOI: 10.1186/s13326-017-0126-0), which also aims to simplify ontology development through table-based templating approaches.

      * Reasonable Ontology Templates (OTTR) framework (Skjæveland & Karlsen, 2024, TGDK 2(2): 5:1-5:54), which provides another template-based approach to ontology construction.

 Including these tools would provide a more comprehensive view of the solution space and help readers understand how OntoSpreadEd's spreadsheet approach compares to template-based methodologies.

 * User Evaluation: The paper would be strengthened by empirical evidence of improved usability compared to existing tools, such as user studies with domain experts or usability metrics.

 * Scalability Considerations: Discussion of how the tool performs with large ontologies or complex hierarchical structures would be valuable for potential adopters.

 * Minor Issues

   * Some sentences could be tightened for clarity (e.g., the sentence beginning "Another typical error occurs when...")

   * The validation features list could be better organized, perhaps as a bulleted list for improved readability

 * Recommendation

 This paper presents a valuable contribution to making ontology development more accessible to domain experts. The tool addresses a real need in the research community, and the technical approach appears sound. With the addition of discussion comparing OntoSpreadEd to DOS-DP and OTTR approaches, this work would provide readers with a more complete understanding of available solutions for simplified ontology development.

 Recommendation: Accept with minor revisions.

 The requested additions regarding DOS-DP and OTTR frameworks would significantly enhance the paper's contribution to the literature by providing a more comprehensive comparative analysis of accessible ontology development approaches.

Are the conclusions about the tool and its performance adequately supported by the findings presented in the article?

Yes

Is the rationale for developing the new software tool clearly explained?

Yes

Is the description of the software tool technically sound?

Yes

Are sufficient details of the code, methods and analysis (if applicable) provided to allow replication of the software development and its use by others?

Yes

Is sufficient information provided to allow interpretation of the expected output datasets and any results generated using the tool?

Yes

Reviewer Expertise:

biomedical ontologies; biomedical data; genetics; molecular biology

I confirm that I have read this submission and believe that I have an appropriate level of expertise to confirm that it is of an acceptable scientific standard.

## References

[1] : Dead simple OWL design patterns. *Journal of Biomedical Semantics* .2017;8(1) : 10.1186/s13326-017-0126-0 10.1186/s13326-017-0126-0 28583177 PMC5460348

[2] Martin G. Skjæveland and Leif Harald Karlsen. The Reasonable Ontology Templates Framework. Transactions on Graph Data and Knowledge (TGDK) Volume 2, Issue 2. TGDK 2(2): 5:1-5:54 (2024).

# Response

**Published:** 2026-07-06

**Authors:** Björn Gehrke

**Affiliations:** Idiap Research Institute, Martigny, Valais, Switzerland

We want to thank you for the constructive feedback and comments on our manuscript. Your contribution has greatly helped us make the manuscript not only clearer but also more contextualised. Below, please find a point-by-point reply to the comments we have received. *Incomplete tool coverage in the comparative analysis of the existing tools: While the comparison with existing tools is valuable, the paper would benefit from discussing additional relevant approaches:* *Dead Simple OWL Design Pattern (DOS-DP) exchange format (Osumi-Sutherland et al., 2017, DOI: 10.1186/s13326-017-0126-0), which also aims to simplify ontology development through table-based templating approaches.* *Reasonable Ontology Templates (OTTR) framework (Skjæveland & Karlsen, 2024, TGDK 2(2): 5:1-5:54), which provides another template-based approach to ontology construction.* *Including these tools would provide a more comprehensive view of the solution space and help readers understand how OntoSpreadEd's spreadsheet approach compares to template-based methodologies.*

 Thank you for this insightful comment. We have now integrated these two approaches into the manuscript.**

*User Evaluation: The paper would be strengthened by empirical evidence of improved usability compared to existing tools, such as user studies with domain experts or usability metrics.*

 Thank you for this comment. We agree that formal usability testing will be useful in improving the tool.

 So far, we have conducted internal user testing with members of a number of projects that are using the tool (BCIO, AddictO, GMHO and TSTO) and the tool has been iteratively improved based on the received user feedback. This has been noted in the requirements section of the manuscript and we noted the need for further extensive user testing in the future work section of the manuscript.**

*Scalability Considerations: Discussion of how the tool performs with large ontologies or complex hierarchical structures would be valuable for potential adopters. * Thank you for this comment. We added a discussion about scalability. *Some sentences could be tightened for clarity (e.g., the sentence beginning "Another typical error occurs when...")*

 We have revised the text for clarity.**

*The validation features list could be better organized, perhaps as a bulleted list for improved readability*

 Thank you for this suggestion. We have changed it into a bulleted list.
